# Fatal Gastric Perforation Caused by Undiagnosed Trichobezoar in an Adolescent: A Case Report

**DOI:** 10.5811/cpcem.52931

**Published:** 2026-04-21

**Authors:** Mert Gültekin, Ayça Erinmez, Yunus Emre Karpuz

**Affiliations:** * Şanlıurfa Training and Research Hospital, Department of Emergency Medicine, Şanlıurfa, Türkiye; † Gaziantep University Faculty of Medicine, Department of Pediatric Surgery, Gaziantep, Türkiye; ‡ Gaziantep University Faculty of Medicine, Department of Emergency Medicine, Gaziantep, Türkiye

**Keywords:** trichobezoar, gastric perforation, cardiac arrest, adolescent, case report

## Abstract

**Introduction:**

Trichobezoar is a rare gastrointestinal condition typically caused by ingestion of hair, which most often affects adolescent females. Its clinical presentation is frequently nonspecific, with symptoms such as abdominal pain, constipation, or early satiety, which can delay recognition until severe complications such as obstruction or perforation develop.

**Case Report:**

We present the case of a 14-year-old girl who developed a massive trichobezoar resulting in gastric perforation and death. She had a three-month history of intermittent constipation and multiple healthcare visits without definitive diagnosis. On arrival to the emergency department, she was in cardiopulmonary arrest. Computed tomography revealed a large intragastric mass with associated pneumoperitoneum. Emergency laparotomy confirmed a trichobezoar with gastric perforation and diffuse peritonitis. Despite prompt surgical and resuscitative intervention, the patient could not be revived.

**Conclusion:**

This case underscores the importance for emergency physicians to maintain clinical vigilance when adolescents present with persistent, unexplained gastrointestinal symptoms. Trichobezoar should be considered in the differential diagnosis, even in the absence of psychiatric history. A low threshold for advanced imaging is warranted, as early recognition and intervention may prevent fatal complications such as gastric perforation and septic shock.

## INTRODUCTION

Bezoars are solid masses formed by indigestible foreign materials within the gastrointestinal (GI) system. The taxonomy of bezoars reflects their diverse origins, with phytobezoars arising from undigested plant matter, trichobezoars developing from accumulated hair fibers, lactobezoars forming from milk protein precipitates, and pharmacobezoars resulting from medication deposits.^1^–^3^ Trichobezoars constitute a small subset of GI bezoars, representing approximately 6% of documented bezoar cases in the medical literature.^1^,^2^ It typically occurs in adolescent girls, particularly those with developmental disabilities and underlying psychiatric disorders. Trichobezoars are associated with trichotillomania, a compulsive disorder characterized by repetitive hair-pulling behavior that causes significant distress, which is classified under obsessive-compulsive and related disorders in the *Diagnostic and Statistical Manual of Mental Disorders*, 5^th^ Edition.^1^,^5^,^6^ Additional precipitating factors include depression, anxiety, neglect, and sudden emotional disruptions within the family.

Trichobezoars may present with nonspecific symptoms such as abdominal pain, constipation, bloating, and nausea, and their clinical presentation may simulate common GI pathologies including functional constipation, gastritis, peptic ulceration, or even abdominal tuberculosis, creating diagnostic uncertainty. Without timely recognition, these masses can cause various complications including protein-losing enteropathy, obstructive jaundice, intussusception, intestinal obstruction, and even gastric perforation, with potential for fatal outcomes.^3^,^4^,^6^,^7^ We present a fatal case involving a 14-year-old girl who developed gastric perforation secondary to a large trichobezoar. Despite multiple emergency department (ED) visits over a three-month period with GI complaints, trichobezoar was never considered in the differential diagnosis. This case demonstrates the diagnostic challenges of adolescent patients and emphasizes the necessity for comprehensive clinical assessment when standard treatments repeatedly fail.

## CASE REPORT

A 14-year-old adolescent girl with no known chronic medical issues was brought to the ED by her family after a sudden collapse at home. On arrival, the patient was unresponsive and in cardiopulmonary arrest. She appeared pale and cyanotic, with no spontaneous respirations and no palpable pulse. Her Glasgow Coma Scale was three. Advanced Life Support was initiated immediately. Endotracheal intubation and chest compressions were performed, and due to difficulty obtaining peripheral intravenous access, an intraosseous line was placed in the right tibia for medications including epinephrine. Return of spontaneous circulation was achieved at the fifteenth minute of resuscitation. Post-resuscitation examination noted fixed, dilated pupils unreactive to light. Point-of-care ultrasound revealed substantial free intraperitoneal fluid in the abdomen (measuring up to 130 mm in depth), and focused cardiac ultrasound showed normal cardiac contractility and no pericardial effusion.

Once the patient was stabilized, further history was obtained from the family. Medical, family, and psychosocial history was unremarkable. No chronic medical conditions, psychiatric disorders, or unusual behavioral patterns were reported by the family. When specifically questioned, the family denied any history of hair-pulling, hair-chewing, or pica-related behaviors. They reported that the patient had experienced intermittent abdominal pain and intractable constipation for the prior three months. During that period, she had been evaluated at multiple healthcare facilities. Each time, her symptoms were managed conservatively or symptomatically, with laxatives, enemas, or herbal remedies, but no definitive diagnosis was established. Notably, just two hours prior to presentation at our hospital, she had been seen at another ED for worsening abdominal discomfort and was given a large-volume enema, with no imaging performed. On our examination, the patient’s abdomen was markedly distended and firm. Bowel sounds were absent. There were no external signs of acute trauma. Genitourinary exam was unremarkable for injury.

*CPC-EM Capsule*

What do we already know about this clinical entity?

*Trichobezoars are gastric masses formed from ingested hair, typically affecting adolescent females with psychiatric comorbidities*.

What makes this presentation of disease reportable?

*In this fatal case of gastric perforation in an adolescent the diagnosis was missed despite recurrent emergency department visits, due to diagnostic anchoring*.

What is the major learning point?

*Recurrent, nonspecific abdominal pain in adolescents warrants advanced imaging and consideration of bezoars, even without known psychiatric history*.

How might this improve emergency medicine practice?

*Recognizing specific red flags and using computed tomography prevents diagnostic delays and catastrophic complications in cryptic abdominal pain cases*.

Laboratory studies revealed a severe inflammatory and metabolic derangement consistent with abdominal sepsis: C-reactive protein, 331 milligrams per liter (mg/L) (reference range: 0–5 mg/L); markedly elevated white blood cell count, 17,350/mm^3^ (5,000–10,000/mm^3^); leukocytosis; hemoglobin 9.6 grams per deciliter (g/dL) (12–16 g/dL); anemia, and metabolic panel notable for creatinine 2.0 mg/dL (0.5–1.1 mg/dL); acute kidney injury; sodium 128 millimoles (mmol)/L (135–145 mmol/L); and potassium 5.8 mmol/L (3.5–5 mmol/L). Arterial blood gas demonstrated a high anion-gap metabolic acidosis, pH 6.70 (7.35–7.45); bicarbonate, 5.9 mmol/L (21.8–26.2); and lactate, 17 mmol/L (0.5–1.6), consistent with profound shock.

**Image 1:**
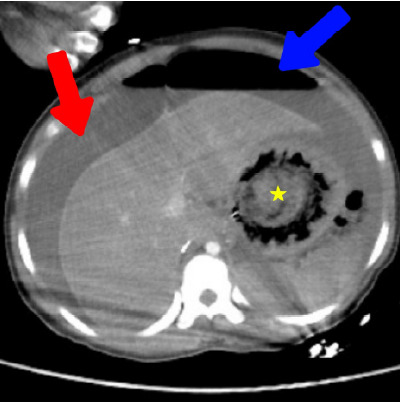
Abdominal computed tomography (axial plane) image at the level of the liver and stomach. Free intraperitoneal fluid (red arrow) and pneumoperitoneum (blue arrow) are present. A large intragastric mass consistent with a bezoar is seen (yellow star).

**Image 2:**
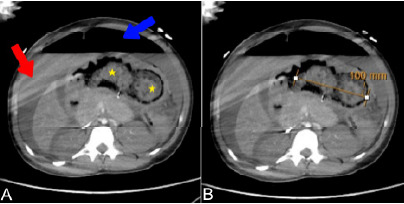
Abdominal computed tomography (axial plane) images at the level of the gastric outlet. (A) Free fluid (red arrow), pneumoperitoneum (blue arrow), and a large intragastric mass (yellow stars) are visible. (B) Radiological measurement of the intragastric mass demonstrates a diameter of approximately 100 millimeters.

Computed tomography (CT) was obtained. Head and chest CT were unremarkable; however, abdominal CT revealed massive pneumoperitoneum and free fluid throughout the peritoneal cavity (Image 1). Impressively, a large bezoar-like foreign body was visible, occupying the stomach and extending through the duodenum, measuring approximately 100 mm at its widest diameter on radiological assessment (Image 2). A full thickness defect was noted along the greater curvature of the stomach, consistent with gastric perforation, and multiple dilated small bowel loops with air-fluid levels suggested secondary intestinal obstruction by the mass.

**Image 3:**
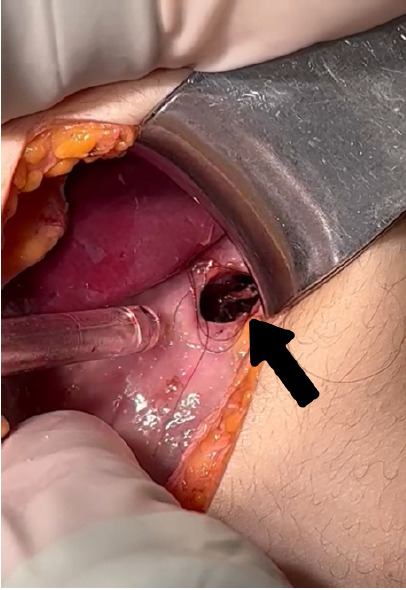
The intraoperative image shows a perforation area (arrow) on the anterior stomach wall, where hair fibers are also present.

Surgical and pediatric specialty consultations were obtained immediately. The patient was started on broad-spectrum antibiotics and taken emergently to the operating theater for exploratory laparotomy. During surgery, a large trichobezoar was found filling the stomach. A perforation approximately 2 cm in diameter was confirmed in the gastric wall at the site where the trichobezoar had likely pressed and eroded through (Image 3). Gross spillage of intestinal contents and pus was present in the peritoneal cavity, consistent with diffuse peritonitis. However, during the procedure the patient suffered a second cardiac arrest, likely due to refractory septic shock. Despite immediate resuscitative efforts, the patient could not be revived and was pronounced dead in the operating room. In accordance with ethical protocols and out of respect for the deceased patient, the surgical intervention was discontinued at that point, and complete trichobezoar removal was not undertaken.

## DISCUSSION

Trichobezoars are rare but serious conditions. They mostly affect teenage girls and are linked to hair-pulling and eating hair.^1^,^5^ Even though this condition is known, it is hard to diagnose early because symptoms are vague and there is often no clear mental health history. Several fatal trichobezoar cases have been reported in the literature.^2^,^8^,^9^ The main problem in diagnosing early is the unclear symptoms. Patients often have general complaints like stomach pain and constipation. In a study of 21 children, Wang et al^2^ reported that stomach pain (90.5%) and vomiting (76.2%) were common symptoms. Mirza et al^4^ saw these symptoms in 88% of 17 cases.

A key sign of trichobezoar is a firm, movable lump in the upper stomach area, known as the Lamerton sign.^10^ The presence of this lump varies; Wang et al 2 reported it in 61.9% of patients and Mirza et al^4^ in 41%. Without a known mental health history, these signs can be misunderstood. Wang et al noted that lumps were wrongly diagnosed as stool or normal stomach contents in four patients.^2^ Gomez-Suarez warned that telling the difference between physical and functional causes based on signs alone is often unreliable.^11^ Obtaining a history of eating hair is hard. Mewa Kinoo and Singh^10^ reported that patients often hide this behavior due to shame or not knowing, making the history “not easily forthcoming.” Therefore, not having a reported mental health history should not rule out the diagnosis.

Relying on traditional “red flags” like bleeding and weight loss for advanced imaging can be misleading. Delin and Berglund^12^ showed that while red flags are good for inflammatory bowel disease, they are not very sensitive (59.4%) for other conditions. Adeniyi et al^13^ found that among children with repeated stomach pain, only 12.6% showed warning symptoms, but 87.4% had identifiable physical problems. Our case demonstrates this issue; the lack of “classic” warning symptoms gave false reassurance, causing deadly delays.

For imaging, ultrasound is the first choice in a young patient to limit exposure to radiation, but it has limitations.^14^ Elghazeery and Hassan^15^ noted that hair’s high echogenicity and shadowing can hide the diagnosis or be mistaken for gas. Thus, if ultrasound is unclear, especially with a lump or repeated symptoms, CT is needed, as it is superior for a clear diagnosis.^2^,^14^ The critical red flags and indications for advanced imaging in adolescent patients with recurrent abdominal pain are summarized in the Table.

This case report has several strengths and limitations that warrant discussion. The primary strength lies in the detailed documentation of a rare but fatal presentation of trichobezoar, providing important educational value for emergency physicians as well as pediatricians and surgeons. The timeline illustrates the diagnostic challenges encountered across multiple healthcare settings, highlighting how nonspecific presentations can obscure recognition of life-threatening conditions in the ED. This report also emphasizes key considerations for children who may present repeatedly with refractory GI complaints, reminding emergency clinicians to re-evaluate prior assumptions when symptoms persist. However, several limitations must be acknowledged. The retrospective nature of the report restricts full reconstruction of the decision-making process during prior healthcare encounters. The absence of detailed documentation from earlier visits prevents comprehensive analysis of missed opportunities for earlier diagnosis.

## CONCLUSION

This case highlights critical lessons for clinical practice, particularly for **emergency physicians** who encounter adolescents with non-specific abdominal complaints. Trichobezoar should be considered in the differential diagnosis of persistent or recurrent gastrointestinal symptoms unresponsive to conventional therapy, even in the absence of psychiatric or behavioral history. Emergency clinicians should maintain a low threshold for advanced imaging when standard treatments fail, as early recognition and intervention are essential to prevent perforation and other fatal complications. More broadly, this case underscores the need for systematic diagnostic approaches in the emergency setting, moving beyond repeated symptomatic management to uncover rare but life-threatening causes of abdominal pain in pediatric patients.

## Figures and Tables

**Table:** Clinical “Red Flags” and Indications for Escalating to Advanced Imaging in Adolescents with Recurrent Abdominal Pain

Clinical Domain	Red Flag / Indication for CT	Rationale & Supporting Literature
History & Course	Recurrent ED visits without definitive diagnosis	Persistent symptoms despite conservative management suggest missed pathology, necessitating re-evaluation beyond standard algorithms. 11,13
Psychiatric History	History of trichotillomania / trichophagia	Strong predictor of trichobezoar (frequently associated with underlying psychiatric disorders), warranting immediate imaging. 10, 15 Note: Absence of a known psychiatric history does not exclude bezoar diagnosis.
Physical Exam	Palpable abdominal mass / Firmness	A firm, mobile epigastric mass (Lamerton’s sign) is a cardinal sign but is frequently misdiagnosed as fecal loading (fecaloma). Unlike fecalomas, bezoars do not resolve with laxative therapy. 10,15
Diagnostic Reliability	Absence of “Classic” Alarm Symptoms	“Classic” red flags (e.g., fever, bleeding) have low sensitivity (<60%) for general organic diseases. Their absence should not preclude imaging. 11,12
Imaging Protocol	Non-diagnostic or Equivocal Ultrasound	Ultrasound is first-line to avoid radiation, but diagnostic uncertainty or limited visualization due to gas/mass requires escalation to CT. 14

*CT*, computed tomography; *ED*, emergency department.
